# H3N2 Mismatch of 2014–15 Northern Hemisphere Influenza Vaccines and Head-to-head Comparison between Human and Ferret Antisera derived Antigenic Maps

**DOI:** 10.1038/srep15279

**Published:** 2015-10-16

**Authors:** Hang Xie, Xiu-Feng Wan, Zhiping Ye, Ewan P. Plant, Yangqing Zhao, Yifei Xu, Xing Li, Courtney Finch, Nan Zhao, Toshiaki Kawano, Olga Zoueva, Meng-Jung Chiang, Xianghong Jing, Zhengshi Lin, Anding Zhang, Yanhong Zhu

**Affiliations:** 1 Laboratory of Respiratory Viral Diseases, Division of Viral Products, Office of Vaccines Research and Review, Center for Biologics Evaluation and Research, United States Food and Drug Administration (CBER/FDA), 10903 New Hampshire Ave, Silver Spring, MD 20993; 2 Department of Basic Sciences, College of Veterinary Medicine, Mississippi State University, 240 Wise Center, Mississippi State, MS 39762.

## Abstract

The poor performance of 2014–15 Northern Hemisphere (NH) influenza vaccines was attributed to mismatched H3N2 component with circulating epidemic strains. Using human serum samples collected from 2009–10, 2010–11 and 2014–15 NH influenza vaccine trials, we assessed their cross-reactive hemagglutination inhibition (HAI) antibody responses against recent H3 epidemic isolates. All three populations (children, adults, and older adults) vaccinated with the 2014–15 NH egg- or cell-based vaccine, showed >50% reduction in HAI post-vaccination geometric mean titers against epidemic H3 isolates from those against egg-grown H3 vaccine strain A/Texas/50/2012 (TX/12e). The 2014–15 NH vaccines, regardless of production type, failed to further extend HAI cross-reactivity against H3 epidemic strains from previous seasonal vaccines. Head-to-head comparison between ferret and human antisera derived antigenic maps revealed different antigenic patterns among representative egg- and cell-grown H3 viruses characterized. Molecular modeling indicated that the mutations of epidemic H3 strains were mainly located in antibody-binding sites A and B as compared with TX/12e. To improve vaccine strain selection, human serologic testing on vaccination-induced cross-reactivity need be emphasized along with virus antigenic characterization by ferret model.

In fall 2014, increased influenza activity was observed in all U.S. regions. By January 17, 2015, pneumonia- and influenza-associated mortality had surpassed the epidemic threshold for that week by 2.2% (http://www.cdc.gov/flu/weekly/weeklyarchives2014-2015/week2.htm#S2). Accompanying this elevated influenza activity was the disappointing performance of 2014–15 Northern Hemisphere (NH) influenza vaccines. Vaccine effectiveness (VE) against laboratory-confirmed influenza associated with medically attended acute respiratory illness was estimated overall at 23% (95% confidence interval [CI], 8–36%)^1^; VE against subtype H3–specific influenza, which represented most cases, was 22% (95% CI, 5–35%)^1^.

Low effectiveness of 2014–15 NH influenza vaccines has been attributed to mismatch of the H3 component with the circulating influenza A (H3) viruses. The majority of H3 isolates characterized were antigenically and genetically distinguished from A/Texas/50/2012 (TX/12), the prototype strain for the H3 component in 2014–15 NH influenza vaccines^2^. Most emerging H3 viruses belonged to antigenic groups 3C.2a and 3C.3a and were antigenically close to A/Switzerland/9715293/2013 (SWZ/13), the H3 strain selected for 2015 Southern Hemisphere (SH) and 2015–16 NH vaccines (http://www.who.int/influenza/vaccines/virus/en/)^1^,^2^,^3^.

Vaccine strain updates require a complex evaluation process, in which the main determinant is antigenic characterization of circulating viruses by standard ferret antisera; lesser determinants are genetic variations, prevalence rates, and geographic distributions of virus variants^4^,^5^. In this process, standard ferret post-infection antisera are obtained by inoculating seronegative ferrets with reference viruses representing recent and emergent influenza isolates. Because influenza viruses are apt to acquire host-mediated mutation(s) at receptor-binding site (RBS) resulting in antigenic changes^6^, usually reference viruses propagated in both mammalian cell- and embryonated egg in parallel are used to generate standard reference ferret antisera. Cross-reactivity of standard ferret antisera to the variant viruses is then determined by hemagglutination inhibition (HAI) assay; a ≥8-fold reduction, compared with the reactivity of standard ferret antisera to homologous vaccine virus, indicates antigenic distinction between new variants and vaccine strains. Antigenic cartography is used to illustrate the relative antigenic relationship among a large number of viruses tested each season^4^,^5^,^7^. In addition, serologic testing is conducted bi-annually to evaluate how well human post-vaccination sera cross-react with representative variants^4^. If there is >50% reduction in post-vaccination response to circulating viruses from those of vaccine strains, it suggests that existing vaccine is inefficient to induce adequate cross-reactive antibodies to neutralize emergent variants^8^. During vaccine strain selection, the cross-reactivity of human post-vaccination sera is used only to confirm representative variants including vaccine candidates selected by antigenic characterization by ferret post-infection antisera. Once vaccine strains are decided, it takes approximately 6 months to manufacture and distribute seasonal vaccines. In most seasons, vaccine strains chosen by this process have matched well with emerging variants. However, a suboptimal match or mismatch can occur, resulting in reduced VE.

The surveillance and VE estimates suggest TX/12 is a mismatch with H3 strains emerging in the United States during the 2014–15 influenza season^1^,^2^. We thus evaluated H3 cross-reactive HAI antibodies induced by egg- or cell-produced 2014–15 NH seasonal influenza vaccines in healthy subjects representing older adults, adults and children. We also compared the HAI cross-reactivity of 2009–10, 2010–11, and 2014–15 NH seasonal influenza vaccines against recently circulating H3 viruses. It has been suggested that ferret may not be an appropriate model to predict antigenic changes for influenza vaccine strain selection^8^,^9^. However, it is yet unclear how different ferret system is from humans and why the differences occasionally lead to a mismatch vaccine strain, etc. These questions often cause confusions not only to the scientific community in different disciplines but also to the public. Using representative H3 viruses circulating during 2007–2014 as examples, we conducted head-to-head comparison on the antigenic maps derived from human and ferret serologic data, and illustrated the differences in antigenic characterization by these two systems. Our model demonstrated that sometimes a mismatch is unavoidable despite the best efforts have been made in seasonal vaccine strain selection. We also discussed the potential factors accounting for the differences observed above.

## Methods

### Ethics statement

All human samples were analyzed anonymously at CBER/FDA.

### Human post-vaccination sera

All clinical protocols were approved by CBER/FDA and the methods were carried out in accordance with the approved guidelines. Written informed consents were provided by subjects at enrollment. Serum samples were collected from healthy subjects before and after vaccination with inactivated NH 2009–10, 2010–11, or 2014–15 egg-based vaccine or 2014–15 cell-based vaccine. There were 24 pairs of sera each from adults (18 to <65 years) vaccinated with 2009–10 and 2010–11 NH egg-based vaccines. There were also 24 pairs of sera each from adults (18 to <65 years) and older adults (≥65 years) administered with 2014–15 NH cell-based vaccine. Sera collected from healthy subjects vaccinated with 2014–15 NH egg-based vaccine include 52 pairs of sera from children (6 months to <9 years), and 30 pairs of sera each from adults (18 to <65 years) and older adults (≥65 years). The demographic characteristics of enrolled vaccinees and their HAI responses against prototype vaccine viruses are presented in Supplementary Tables S1–S4.

### Viruses

The following egg-grown viruses were propagated in 9- to 10-day-old specific pathogen–free embryonated eggs: H1N1 strains A/Brisbane/59/2007 and A/California/07/2009, H3N2 strains A/Uruguay/716/2007 (URY/07e), A/Perth/16/2009 (PE/09e), A/Victoria/361/2011 (VIC/11e), TX/12e, A/Costa Rica/4700/2013 (CRI/13e), A/Utah/07/2013 (UT/13e), A/Switzerland/9715293/2013 (SWZ/13e), A/Palau/6759/2014 (PL/14e), A/North Carolina/13/2014 (NC/14e), and B/Brisbane/60/2008. The following cell-grown viruses were amplified in Madin-Darby kidney cells: H3N2 strains VIC/11c, TX/12c, CRI/13c, UT/13c, SWZ/13c, NC/14c, A/Michigan/15/2014 (MI/14c), and B/Texas/02/2012. Except for reassortant URY/07e, all strains were wild-type viruses with the hemagglutinin (HA) confirmed by full-length DNA sequencing.

### HAI assay

Before being assayed, serum samples were pretreated with receptor-destroying enzyme (Denka-Seiken). Turkey erythrocytes (0.5%) for influenza H1N1 and B viruses, or guinea pig erythrocytes (1%) for H3N2 viruses were used in HAI assays, as described^10^,^11^. HAI titers were expressed as the reciprocal of the highest serum dilution that resulted in complete inhibition of hemagglutination. A titer of 5 was assigned if no inhibition was observed at the starting 1:10 serum dilution. Unless otherwise specified, human and ferret HAI titers were determined on the same day, using the same virus batches and erythrocyte preparations. Standard ferret post-infection antisera were obtained from Centers for Diseases Control and Prevention (CDC) and were pooled from 3 or 4 infected ferrets with similar post-infection HAI titers. Ferret cross-reactive HAI titers against recent H3 isolates in the testing panel are summarized in Supplementary Table S5.

### Data analysis and statistics

We determined geometric mean titers (GMTs) for pre-vaccination and post-vaccination HAI titers as well as seroprotection rates (% with post-vaccination HAI titer ≥40) and seroconversion rates (% with 4-fold increase of HAI titer when either a pre-vaccination HAI titer ≤1:10 and a post-vaccination HAI titer ≥1:40, or a pre-vaccination HAI titer >1:10 and a minimum 4-fold rise in post-vaccination HAI antibody titer). Statistical analysis was performed using log-transformed HAI titers with ratio paired *t*-test and two-tailed *p* value with GraphPad Prism (version 6.05). *p* ≤ 0.05 was considered statistically significant.

### Antigenic cartography and molecular modeling

We constructed antigenic maps based on human or ferret HAI data by using AntigenMap (http://sysbio.cvm.msstate.edu/AntigenMap) with an HAI titer of 10 as the cutoff^12^,^13^,^14^,^15^. The post-vaccination sera from adults who had pre-vaccination HAI titers of <40 against all H1, H3, and B viruses tested in the study, defined as H1/H3/B-unprimed, were selected to construct human serology–based antigenic maps. There were 23 H1/H3/B-unprimed adult sera from 2009–10, 2010–11, and 2014–15 NH egg-based vaccine trials, 8 H1/H3/B-unprimed adult sera from 2014–15 NH cell-based vaccine trial, and a total of 31 samples from the combined trials with egg- and cell-based vaccinations. The three-dimensional hemagglutinin 1 (HA1) structures of TX/12e and SWZ/13e were constructed by using the H3 HA1 template (Protein Data Bank code 4WE8) and homology modeling with MODELLER^16^,^17^. Antibody-binding sites (ABS) and receptor-binding sites (RBS) were adapted from previous annotations^18^,^19^.

## Results

### H3N2-specific HAI cross-reactivity of 2014–15 NH vaccines

**Figure 1:**
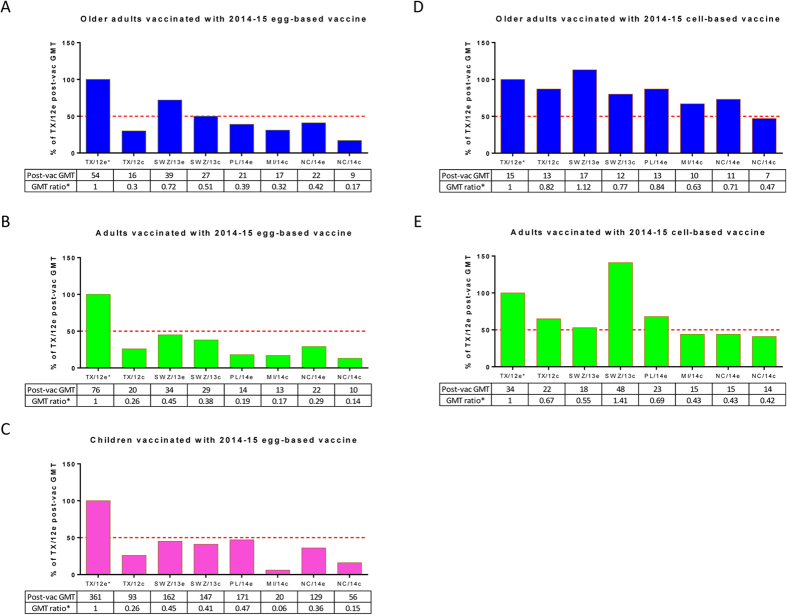
Cross-reactive hemagglutination inhibition (HAI) responses against the latest epidemic influenza A (H3) isolates in humans vaccinated with the 2014–15 Northern Hemisphere (NH) seasonal influenza vaccines. Sera were collected from healthy children (6 months to <9 years), adults (18 to <65 years), and older adults (≥65 years), vaccinated with 2014–15 NH egg- or cell-based vaccine. Post-vaccination (post-vac) HAI titers against the H3 vaccine prototype virus and representative isolates of the latest H3N2 epidemic strains were determined by using 1% guinea pig erythrocytes. There were 30 post-vac sera each from adult and older adult populations and 52 post-vac sera from children administered 2014–15 NH egg-based vaccine. There were also 24 post-vac sera each from adult and older adult populations administered 2014–15 NH cell-based vaccine. The H3 strains in the testing panel were as follows: egg-grown A/Texas/50/2012 (TX/12e), A/Switzerland/9715293/2013 (SWZ/13e), A/Palau/6759/2014 (PL/14e), A/North Carolina/13/2014 (NC/14e), and cell-grown A/Texas/50/2012 (TX/12c), A/Switzerland/9715293/2013 (SWZ/13c), A/North Carolina/13/2014 (NC/14c), and A/Michigan/15/2014 (MI/14c). *Indicates the H3 prototype virus of 2014–15 NH egg-based vaccine. Cross-reactive post-vac geometric mean titers (GMTs) against testing H3 viruses are expressed as % of TX/12e post-vac GMT in the bar graphs. Numbers shown at the bottom of each bar graph are individual post-vac GMTs and GMT ratios relative to TX/12e. Red dashed horizontal line indicates 50% of TX/12e-specific GMT.

Overall, most subjects administered egg- or cell-based 2014–15 NH vaccines achieved acceptable HAI titers for each vaccine component, indicating successful immunization (Supplementary Tables S1 and S2). However, vaccination with 2014–15 NH egg-based vaccine yielded substantially lower HAI titers toward recent H3 variants, particularly cell-grown strains, than toward H3 vaccine component TX/12e (Fig. 1A–C). In adults and children, post-vaccination GMTs against recent H3 variants were all <50% of those against TX/12e (GMT ratios <0.5, *p*<0.001; Fig. 1B,C). In general, a >50% reduction in new isolate specific GMT from that of existing vaccine strain means current vaccine is unable to induce sufficient cross-reactive antibodies to well neutralize emergent variants^20^. Compared with egg-based vaccine, the 2014–15 NH cell-based vaccine was less immunogenic, yielding consistently lower GMTs against all egg- and cell-grown H3 strains tested (Fig. 1D,E). GMTs against recent MI/14c, NC/14e, and NC/14c were all >50% lower than that against TX/12e in adults administered cell-based vaccine (Fig. 1E). In general, the 2014–15 NH vaccines, regardless of production type, exhibited limited cross-reactive HAI responses to circulating H3N2 variants.

### H3N2-specific HAI cross-reactivity of previous vaccines

**Figure 2:**
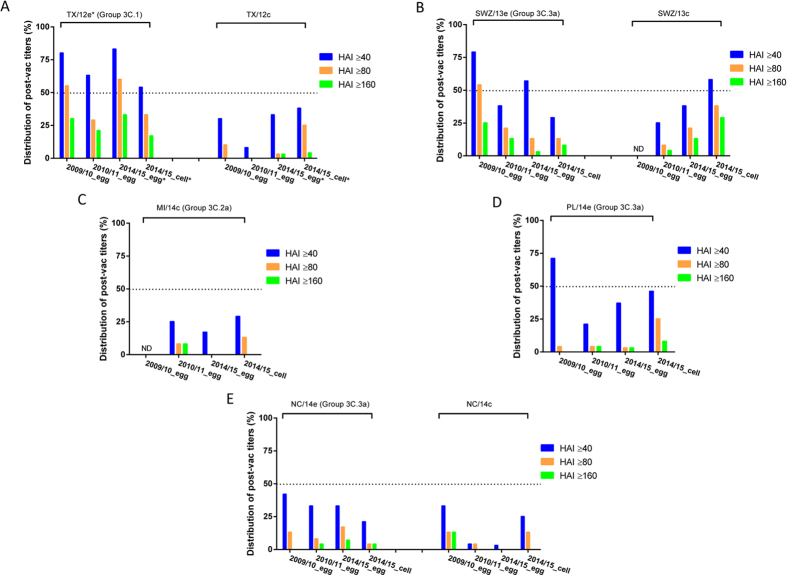
Cross-reactive hemagglutination inhibition (HAI) responses of 2009–10, 2010–11, and 2014–15 Northern Hemisphere (NH) seasonal influenza vaccines against epidemic influenza A (H3) viruses. Serum samples were collected from healthy adults immunized with 2009–10, 2010–11, and 2014–15 NH egg-based or cell-based vaccine. Post-vaccination (post-vac) HAI titers against the H3 vaccine prototype virus and representative isolates of the latest H3N2 epidemic strains were determined by using 1% guinea pig erythrocytes. There were 24 post-vac sera each from adults vaccinated with 2009–10 and 2010–11 NH egg-based vaccines and 2014–15 NH cell-based vaccine. There were also 30 post-vac sera from adults vaccinated with 2014–15 NH egg-based vaccine. The H3 strains in the testing panel included egg-grown A/Texas/50/2012 (TX/12e), A/Switzerland/9715293/2013 (SWZ/13e), A/Palau/6759/2014 (PL/14e), A/North Carolina/13/2014 (NC/14e), and cell-grown A/Texas/50/2012 (TX/12c), A/Switzerland/9715293/2013 (SWZ/13c), A/North Carolina/13/(NC/14c), and A/Michigan/15/2014 (MI/14c). The proportions of subjects with post-vac HAI titer of ≥40, ≥80 and ≥160 were plotted. *Indicates the H3 prototype virus of 2014–15 NH egg-based vaccine. Dotted horizontal line indicates 50% achievement. ND: not determined due to limited volumes of sera.

We compared H3-specific cross-reactive HAI antibodies in archived adult sera from 2009–10 and 2010–11 NH egg-based vaccines with those from 2014–15 NH egg- and cell-based vaccines (Fig. 2; Supplementary Figure S1; Supplementary Table S6). Adults administered 2009–10 egg-based vaccine demonstrated a relatively broad cross-reactive HAI response: post-vaccination GMTs and seroprotection rates were comparable to or exceeded those induced by 2014–15 egg- or cell-based vaccines against recent H3 isolates (Fig. 2A–E; Supplementary Figure S1D and S1E; Supplementary Table S6). Seroprotection rates for adults administered 2009–10 versus 2014–15 egg-based vaccine were 79% versus 57% for SWZ/13e, 71% versus 37% for PL/14e, 42% versus 33% for NC/14e, and 33% versus 3% for NC/14c (Fig. 2B,D,E). Of adults administered 2010–11 egg-based vaccine, 21% to 38% had a seroprotective titer against emergent H3 variants (Fig. 2B–E).

### Antigenic characterization by cartography

**Figure 3:**
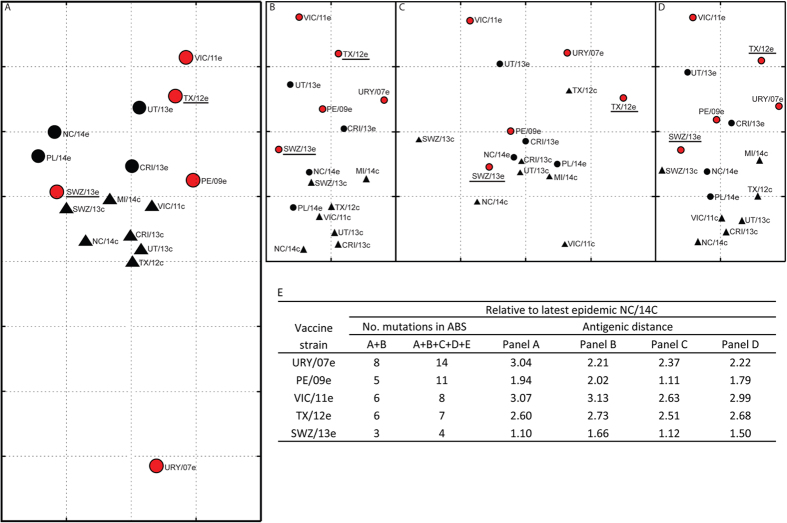
Antigenic characterization of influenza A (H3N2) viruses by cartography. Antigenic maps were constructed on the basis of human hemagglutination inhibition (HAI) or ferret HAI data using AntigenMap (http://sysbio.cvm.msstate.edu/AntigenMap). An HAI titer of 10 was set as the cutoff for negative reaction in the HAI assay. Each entry in the HAI table was normalized by the maximum value from individual serum samples. Noise in the HAI data was minimized by implementing low-rank matrix completion. A two-dimensional map with multidimensional scaling was used to reflect antigenic distances among influenza A (H3) viruses. Each gridline (horizontal and vertical) is one antigenic unit distance corresponding to a 2-fold difference in HAI titers. Dots indicate egg-grown strains A/Uruguay/716/2007 (NYMCX175C) (URY/07e), A/Perth/16/2009 (PE/09e), A/Victoria/361/2011 (VIC/11e), A/Texas/50/2012 (TX/12e), A/Costa Rica/4700/2013 (CRI/13e), A/Utah/07/2013 (UT/13e), A/Switzerland/9715293/2013 (SWZ/13e), A/Palau/6759/2014 (PL/14e), and A/North Carolina/13/2014 (NC/14e). Triangles indicate cell-grown strains A/Victoria/361/2011 (VIC/11c), A/Texas/50/2012 (TX/12c), A/Costa Rica/4700/2013 (CRI/13c), A/Utah/07/2013 (UT/13c), A/Switzerland/9715293/2013 (SWZ/13c), A/North Carolina/13/2014 (NC/14c), and A/Michigan/15/2014 (MI/14c). Red indicates H3 vaccine prototype viruses, and underlining indicates prototype strains TX/12e for 2014–15 NH vaccines and SWZ/13e for 2015–16 NH vaccines. (Panel **A**), ferret post-infection sera–derived map. The post-vaccination sera from adults who had pre-vaccination HAI titers of <40 against all H1, H3, and B viruses tested in the study, defined as H1/H3/B-unprimed, were selected to construct human serology–based antigenic maps. There were 23 H1/H3/B-unprimed adult sera from 2009–10, 2010–11, and 2014–15 NH egg-based vaccine trials (Panel **B**), 8 H1/H3/B-unprimed adult sera from 2014–15 NH cell-based vaccine trial (Panel **C**), and a total of 31 samples from the combined trials with egg- and cell-based vaccinations (Panel **D**). the numbers of mutations in antibody-binding sites (ABS) (**A**–**E**) and antigenic distances relative to NC/14c shown in panel (A–**D**).

To characterize the antigenic relationships among all H3 viruses in the panel, we generated an antigenic map based on ferret HAI data (Fig. 3A; Supplemental Table S5). Results showed that vaccine strains URY/07e (2009–10), PE/09e (2010–11), VIC/11e (2012–13), and TX/12e (2014–15) were antigenically separated by ferret antisera: TX/12e was closer to VIC/11e, and URY/07e was antigenically distinct from all later H3 strains (Fig. 3A). In this ferret map, cell-grown H3N2 strains clustered together and separated from their corresponding egg-grown strains, indicating that the cell-grown H3 viruses were antigenically different from their egg-grown counterparts (Fig. 3A). The ferret antisera-derived map also demonstrated that SWZ/13e (selected for 2015 SH and 2015–16 NH vaccines) was antigenically closer than TX/12e to recent cell-grown H3 strains (Fig. 3A), and the antigenic distance between SWZ/13e and latest epidemic NC/14c was less than half of that between TX/12e and NC/14c (Fig. 3E). In addition, the antigenic distance of NC/14c to recent vaccine strains defined by ferret antisera appeared to correlate with the total mutations from ABS A and B but not from all 5 epitopes combined (Fig. 3E and Supplementary Table S7).

However, ferret antisera induced by primary pandemic H1N1 (pH1N1) infection have been shown to target different HA antigenic sites from human post-infection antisera^8^,^9^, suggesting ferret may not be an optimal model to predict the cross-reactivity of human antibody responses. To better understand how this imperfection sometimes lead to a mismatch of vaccine strain, e.g. TX/12e of 2014–15 NH vaccines, we also used human post-vaccination HAI data shown in this study to construct separate antigenic maps and did head-to-head comparison with the ferret map. Only data for subjects shown in Fig. 2 and Supplementary Figure S1 who had pre-vaccination HAI titers <40 against all H1, H3, and B viruses tested (defined as H1/H3/B-unprimed) were selected to construct human serology–based maps. A total of 23 H1/H3/B-unprimed adult serum samples were available from 2009–10, 2010–11, and 2014–15 NH egg-based vaccine trials (Fig. 3B); 8 were available from the 2014–15 NH cell-based vaccine trial (Fig. 3C) and 31 were available from the combined trials with egg- and cell-based vaccines (Fig. 3D).

In human sera–derived maps, cell-grown strains were antigenically separated from their corresponding egg-grown strains (Fig. 3B–D), which was similar to the ferret map. Of note, cell-grown isolates were more antigenically distinct from their egg-grown counterparts in the map derived from human sera with cell-based vaccine than in the map derived from human sera with egg-based vaccine (Fig. 3B vs. 3C). In the map derived from human sera with cell-based vaccine, TX/12c was even farther away than TX/12e from cell-grown isolates, as compared with their antigenic positions in the map derived from human sera with egg-based vaccine (Fig. 3C vs. 3B). Also unlike the map derived from human sera with egg-based vaccine, VIC/11c was antigenically distinct from the rest of cell-grown isolates in the map derived from human sera with cell-based vaccine (Fig. 3B vs. 3C). In addition, the relative antigenic distance between SWZ/13e and SWZ/13c was much smaller than that between TX/12e and TX/12c in the human sera-derived map with egg-based vaccine as compared to that with cell-based vaccine (Fig. 3B vs 3C). These phenomena indicated that antigenic characterization of epidemic isolates by human serology was likely influenced by the type of vaccine administered.

Unlike the ferret map in which PL/14e and NC/14e of the latest H3 group 3.3a strains were antigenically separated into the group of egg-grown H3 viruses (Fig. 3A), these strains in the human sera–derived maps appeared much closer to cell-grown H3 viruses (Fig. 3B–D). Additionally, SWZ/13e and SWZ/13c were antigenically separated by human post-vaccination sera (Fig. 3B–D) but could not be distinguished by ferret antisera (Fig. 3A). The biggest difference was the antigenic standing of URY/07e, which clustered with egg-grown H3 strains in human sera–derived maps but separated from other H3 viruses in the ferret antisera–derived map (Fig. 3A–D). The antigenic distance between URY/07e and latest epidemic NC/14c was smaller in all human sera-derived maps than in the ferret map (Fig. 3E). Nevertheless, both ferret and human antisera-derived maps exhibited that, SWZ/13e among all vaccine strains was the closest to current epidemic H3 viruses followed by PE/09e; whereas VIC/11e was more distinctly separated (Fig. 3A–E).

### Molecular characterization and modeling

**Figure 4:**
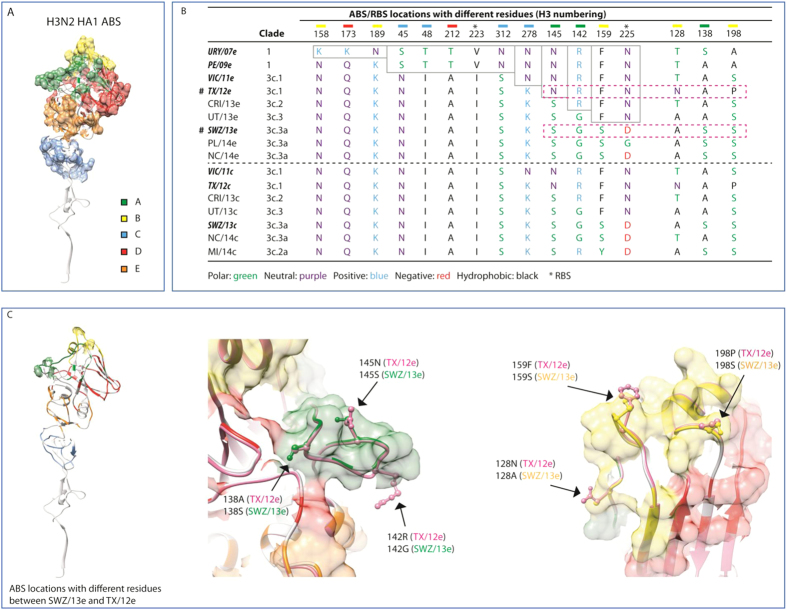
Molecular modeling of influenza A (H3N2) viruses. The three-dimensional structures of hemagglutinin 1 (HA1) of egg-grown A/Texas/50/2012 (TX/12e) and A/Switzerland/9715293/2013 (SWZ/13e) were constructed based on the template PDB ID 4WE8 by homology modeling using MODELLER. Antibody-binding sites (ABS) and receptor-binding site (RBS) were adapted from previous annotations. The changes between TX/12e and SWZ/13e were highlighted at the structural superposition. (Panel **A**) shows the locations of 5 annotated ABS (**A**–**E**) on H3 HA1; (Panel **B**) highlighted mutations at the ABS and RBS among all H3N2 viruses in the testing panel; (Panel **C**) shows structural locations of amino acid differences on ABS between TX/12e and SWZ/12e. The conserved residues are gray-outlined boxes; changes between TX/12e and SWZ/13e are in boxes outlined with magenta-colored dashes. Egg-grown viruses are A/Uruguay/716/2007 (NYMCX175C) (URY/07e), A/Perth/16/2009 (PE/09e), A/Victoria/361/2011 (VIC/11e), A/Costa Rica/4700/2013 (CRI/13e), A/Utah/07/2013 (UT/13e), A/Palau/6759/2014 (PL/14e), and A/North Carolina/13/2014 (NC/14e) in addition to TX/12e and SWZ/13e. Cell-grown strains are A/Victoria/361/2011 (VIC/11c), A/Texas/50/2012 (TX/12c), A/Costa Rica/4700/2013 (CRI/13c), A/Utah/07/2013 (UT/13c), A/Switzerland/9715293/2013 (SWZ/13c), A/North Carolina/13/2014 (NC/14c), and A/Michigan/15/2014 (MI/14c).

HA sequencing results indicated that the differences between TX/12e and SWZ/13e were mainly located at antibody-binding site ABS A and B (Fig. 4A–C; Supplementary Table S7). N145S (A) and P198S (B) consistently occurred in the latest epidemic isolates of HA groups 3C.2a and 3C.3a, regardless of whether egg- or cell-grown (Fig. 4B,C; Supplementary Table S7). N128A (B) and R142G (A) also occurred predominantly in H3 group 3C.3a isolates (Fig. 4B,C; Supplementary Table S7). In addition, an N225D mutation from TX/12e at RBS also predominated in the latest H3 isolates (Fig. 4B). These mutations also highlighted the differences between recent H3 isolates and URY/07e (Fig. 4B; Supplementary Table S7). ABS E appeared to be most conserved. ABS D was conserved to at a lesser extent, exhibiting fewer mutations between URY/07e and the latest H3 isolates (Fig. 4B; Supplementary Table S7).

## Discussion

Seasonal influenza vaccine effectiveness essentially depends upon how well vaccine strains represent viruses circulating in the community. Although a suboptimal vaccine match can provide some protection, mismatched vaccines generally result in reduced protective efficacy^21^,^22^. TX/12e, the H3 component of 2014–15 NH vaccines, matched poorly with most epidemic H3 viruses^2^,^23^, leading to an H3-specific VE of 22%^1^. Study subjects who received 2014–15 NH vaccines had substantially lower levels of HAI antibodies cross-reacting with recent H3 variants than with TX/12e, indicating vaccination-induced antibodies could not efficiently neutralize emergent H3 viruses. The major differences between TX/12e and epidemic H3 isolates are at ABS A and B (Fig. 4; Supplementary Table S7). ABS A and B are constantly under positive selection, and since 1968, they have been responsible for all major antigenic drift during H3 evolution^24^,^25^,^26^,^27^,^28^,^29^,^30^,^31^,^32^. Our study also suggested that the relative antigenic relationships of epidemic viruses characterized by ferret antisera were likely to be impacted more by the mutations arising from both ABS A and B than from all 5 epitopes. This is consistent with the report by Koel *et al*. that antibodies raised in H3N2-infected ferrets are mainly directed against H3 ABS A and B^32^. A single amino acid substitution on seven cluster-transition positions exclusively located in ABS A and B has been found sufficient to cause a dramatic change in H3 antigenicity^7^,^32^. N128A (or N128T) and P198S mutations in ABS B along with position 156 (B), 278 (C), 219 (D) antigenically differentiate TX/12e from VIC/11e, the H3 prototype virus of the 2012–13 influenza season which has also been reported to have low H3N2-specific VE^3^,^33^. In addition, N225D (or N225G) located in RBS along with N128A (or N128T) and N145S could potentially alter HA receptor-binding affinity of recent H3 viruses by causing the loss of *N*-linked glycosylation. Also egg-adapted mutations, such as G186V, in combination with Q156H (B) or L194P (B) could alter the antigenicity of egg-grown H3 viruses^29^,^30^. In this study, cross-reactive HAI responses shown by vaccinees to cell-grown H3 strains were generally lower than responses shown to their egg-grown counterparts.

Phylogenetically TX/12e is closer than URY/07e to the latest H3 viruses with fewer residues different in HA1. The ferret antisera–derived map also showed that URY/07e was antigenically distant from all other H3 viruses tested (Fig. 3A). However, compared with human 2014–15 serum samples, human 2009–10 samples exhibited equal or slightly better HAI cross-reactivity toward recent epidemic H3 viruses (Fig. 2). In human post-vaccination sera–derived maps, URY/07e was antigenically related to TX/12e and was close to recent egg-grown H3 strains, as defined by human sera from combined egg- and cell-based vaccine trials (Fig. 3B,D).

Because of the human-like respiratory physiology of ferrets, ferret post-infection antisera are the reference standards in antigenic characterization for influenza surveillance and vaccine strain selection^4^,^5^,^8^,^28^,^30^,^34^,^35^. Also because of the ultra-sensitivity of ferret post-infection antisera to antigenic changes in influenza viruses, the threshold for warranting a vaccine strain change has been increased from a ≥4-fold to a ≥8-fold reduction in existing vaccine strain–specific HAI titers^5^,^28^. This decision was made to avoid frequent and unnecessary vaccine strain updates.

Despite the similarity of ferret and human respiratory tracts, the systemic process of ferret immunity to influenza infections is unclear because an in-depth understanding of the ferret genome, physiology, and immunology is lacking. Lee *et al*. have reported that HAI cross-reactivity of human sera induced by intranasal vaccination of FluMist (live attenuated influenza vaccine) was distinct from that of ferret post-infection antisera^36^. This suggests human and ferret antibodies even though elicited via the same route are directed to target different epitopes of influenza HA. Indeed, human antibodies specific for pH1N1 viruses are primarily focused on H1 RBS and stalk region^9^,^37^,^38^; whereas ferret anti-pH1N1 antibodies predominantly target the variable Sa antigenic site of H1 HA^39^. Additionally, human H3N2-specific antibodies appear to be directed against either H3 ABS A or B depending upon infecting H3 strains^31^,^40^. In contrast, both H3 ABS A and B are the primary targets of ferret anti-H3N2 antibodies^32^. All these suggest that the process of ferret immunity is different from that of human despite they have similar respiratory tracts.

Also compared with humans, ferrets have a short and clearly defined infection history. To produce standard reference sera for influenza surveillance and vaccine strain selection, only seronegative ferrets that have no prior influenza infections are used^8^. In contrast, human immunity is largely shaped by previous influenza infection and/or vaccination history^9^,^35^,^37^,^38^,^41^. In this study, we chose only human sera that had a pre-vaccination HAI titer of <40 against all H1/H3/B viruses tested to generate antigenic maps. However, it is highly possible that these subjects had prior influenza exposures but their antibody specificity or affinity may not be strong enough to be differentiated by 2-fold serial serum dilutions executed in HAI assays. This might be the reason why the 2009–10 vaccine strain URY/07e appeared much closer to recent H3 viruses in human sera-derived maps than in the ferret map. In fact, the distance of URY/07e to recent H3 isolates was even closer in the antigenic maps derived from all testing human serum samples including H1/H3/B-unprimed as well as H1/H3/B-primed sera (data not shown), suggesting H1/H3/B-primed human sera may be directed against an epitope that is conserved among recent H3 viruses. Li *et al*. have demonstrated that sera collected from pH1N1-infected young adults born between1983–96 primarily targeted an epitope near the stalk region, the homology of which is also shared by previous seasonal H1N1 viruses circulating between1983–96^9^. All these suggest the affinity and specificity of human antibodies are impacted by prior exposure history which is not recapitulated by standard reference ferret sera of primary infection^8^. In addition, our study showed that 2014–15 NH cell-based vaccine was much less immunogenic than 2014–15 NH egg-based vaccine in inducing H3 specific HAI antibodies, despite that both vaccines were produced from the same egg-derived vaccine seed TX/12e. As the result, the antigenic map generated by human sera with cell-based vaccine was different from that with egg-based vaccine, suggesting human HAI cross-reactivity was also influenced by the type of vaccine administered. Cell-based vaccines just recently became available and egg-based vaccines still dominate the supply. Which type of vaccine yields better cross-reactivity is yet to be determined.

Furthermore, ferrets are poor at mounting a detectable HAI response via intramuscular immunization unless an adjuvant is used^42^. Primary ferret post-infection antisera are thus used for vaccine strain selection and characterization. Of note, serum antibody subtype profiles induced by intranasal infection of live viruses differ from those induced by parenteral immunization with inactivated vaccines in ferrets, and adjuvants also change antibody profiles^42^,^43^,^44^. Conceivably, the differences between ferret post-infection sera and human post-vaccination sera would be magnified further by the cross-species differences. As shown in Fig. 3E, human post-vaccination sera, regardless of vaccine type, were less sensitive than ferret post-infection sera to antigenic changes in ABS A and B. Thus, vaccine strains selected on the basis of ferret post-infection sera may not always yield similar cross-reactivity in humans; a mismatch is sometimes inevitable.

Nevertheless, we demonstrate that human post-vaccination sera responded differently than ferret post-infection antisera to recent H3 viruses by head-to-head comparison of the antigenic maps derived. Despite the differences, both ferret and human sera-derived antigenic maps also show that SWZ/13e is antigenically closer than TX/12e to epidemic H3 viruses, indicating that SWZ/13e would be a better match than TX/12e as the vaccine strain for the 2015 SH and 2015–16 NH influenza seasons. Of note, most H3 epidemic strains tested including SWZ/13e are from HA clade 3C.3a, while the majority of H3N2 viruses in circulation since the fall of 2014 are the phylogenetic 3C.2a^23^. Whether SWZ/13e-based 2015 SH and 2015–16 NH vaccines are sufficiently immunogenic as well as efficiently cross-reactive against circulating 3C.2a viruses are currently under investigation. Despite ferret model represents the best strategic approach to detect virus antigenic changes, our data also exemplify its imperfection as a predictive tool for human vaccine efficacy. To improve vaccine strain selection, more emphasis should be put on the role of human serologic testing in assessing vaccination-induced cross-reactivity.

## Additional Information

**How to cite this article**: Xie, H. *et al*. H3N2 Mismatch of 2014-15 Northern Hemisphere Influenza Vaccines and Head-to-head Comparison between Human and Ferret Antisera derived Antigenic Maps. *Sci. Rep*. **5**, 15279; doi: 10.1038/srep15279 (2015).

## Supplementary Material

Supplementary Information
