# Supplementary material for: H3N2 Mismatch of 2014–15 Northern Hemisphere Influenza Vaccines and Head-to-head Comparison between Human and Ferret Antisera derived Antigenic Maps
Source: Sci Rep. 2015 Oct 16;5:15279. doi: 10.1038/srep15279 (PMC4607887; doi:10.1038/srep15279)

H3N2 Mismatch of 2014–15 Northern Hemisphere Influenza Vaccines and Head-to-head Comparison between Human and Ferret Antisera derived Antigenic Maps

Hang Xie[1*], Xiu-Feng Wan[2*], Zhiping Ye[1], Ewan P. Plant[1], Yangqing Zhao[1], Yifei Xu[2], Xing Li[1], Courtney Finch[1], Nan Zhao[2], Toshiaki Kawano[1], Olga Zoueva[1], Meng-Jung Chiang[1], Xianghong Jing[1], Zhengshi Lin[1], Anding Zhang, Yanhong Zhu[1]

[1]Laboratory of Respiratory Viral Diseases, Division of Viral Products, Office of Vaccines Research and Review, Center for Biologics Evaluation and Research, United States Food and Drug Administration (CBER/FDA), 10903 New Hampshire Ave, Silver Spring, MD 20993; [2]Department of Basic Sciences, College of Veterinary Medicine, Mississippi State University, 240 Wise Center, Mississippi State, MS 39762

*Reprint or correspondence: Dr. Hang Xie (Hang.Xie@fda.hhs.gov) and Dr. Xiu-Feng Wan (Wan@cvm.msstate.edu).

Supplementary Table S1. Hemagglutination inhibition responses in healthy subjects administered 2014─15 Northern Hemisphere egg-based seasonal quadrivalent influenza vaccine against homologous vaccine viruses.

		Older adults (n = 30) mean age 72.7 Yr (range, 65.8-84.1 Yr)				Adults (n = 30) mean age 42.7 Yr (range, 18.0-62.4 Yr)				Children (n =5 2) mean age 4.1 Yr (range, 1.1-9.1 Yr)			
Homologous vaccine virus†	Passage	GMT* Pre	GMT* Post	% Seroprotection‡	% Seroconversion§	GMT Pre	GMT Post	% Seroprotection	% Seroconversion	GMT Pre	GMT Post	% Seroprotection	% Seroconversion
pH1N1, CA/07/2009	E6	17	27	53	10	15	39	67	30	47	262	88	56
H3N2, TX/50/2012	E5/E1	24	54	83	33	24	76	83	47	93	361	98	62
B-Vic, B/BR/60/2008	E11	14	24	50	20	14	68	87	67	14	138	81	67
B-Yam, B/MA/02/2012	C4/C1	21	31	53	10	28	80	77	30	17	94	79	63

*GMT: geometric mean titer; Pre, pre-vaccination; Post, post-vaccination.

†The 2014─15 Northern Hemisphere egg-based quadrivalent influenza vaccine contains pandemic H1N1 (pH1N1) A/California/07/2009 (CA/07/2009), H3N2 A/Texas/50/2012-like (TX/50/2012), B-Victoria lineage (B-Vic) B/Brisbane/60/2008 (B/BR/60/2008), and B-Yamagata lineage (B-Yam) B/Massachusetts/02/2012 strains.

‡Percentage with post-vaccination HAI titer ≥40.

§Percentage with 4-fold increase of HAI titer when either a pre-vaccination HAI titer ≤1:10 and a post-vaccination HAI titer ≥1:40 or a pre-vaccination HAI titer >1:10 and a minimum 4-fold rise in post-vaccination HAI antibody titer.

Supplementary Table S2. Hemagglutination inhibition responses in healthy subjects administered 2014─15 Northern Hemisphere cell-based seasonal trivalent influenza vaccine against homologous vaccine viruses.

		Older adults (n = 24), age range ≥ 65 Yr				Adults (n = 24), age range18-64 Yr			
		GMT*				GMT			
Homologous vaccine virus†	Passage	Pre	Post	% Seroprotection‡	% Seroconversion§	Pre	Post	% Seroprotection	% Seroconversion
pH1N1, CA/07/2009	E6	10	22	38	25	20	180	88	75
H3N2, TX/50/2012	E5/E1	9	15	29	13	9	34	54	50
B-Yam, B/MA/02/2012	C4/C1	ND	ND	ND	ND	ND	ND	ND	ND
B-Vic, B/BR/60/2008	E11	9	35	67	46	10	36	58	38

*GMT: geometric mean titer; Pre, pre-vaccination; Post, post-vaccination.

†The 2014─15 Northern Hemisphere cell-based trivalent influenza vaccine contains pandemic H1N1 (pH1N1) A/California/07/2009-like (CA/07/2009), H3N2 A/Texas/50/2012-like (TX/50/2012), and B-Yamagata lineage (B-Yam) B/Massachusetts/02/2012 strains. B-Victoria lineage (B-Vic) B/Brisbane/60/2008 (B/BR/60/2008) was the 2nd B strain included in 2014─15 Northern Hemisphere quadrivalent influenza vaccine.

‡Percentage with post-vaccination HAI titer ≥40.

§Percentage with 4-fold increase of HAI titer when either a pre-vaccination HAI titer ≤1:10 and a post-vaccination HAI titer ≥1:40 or a pre-vaccination HAI titer >1:10 and a minimum 4-fold rise in post-vaccination HAI antibody titer.

ND: not determined due to limited volumes of sera.

Supplementary Table S3. Hemagglutination inhibition responses in healthy subjects administered 2009–10 Northern Hemisphere egg-based seasonal trivalent influenza vaccine against homologous vaccine viruses.

		Adults (n = 24), mean age 44.7 Yr (range, 22.8-64.3 Yr)			
		GMT*			
Homologous vaccine virus†	Passage	Pre	Post	% Seroprotection‡	% Seroconversion§
H1N1, BR/59/2007	E8	5	60	71	71
H3N2, URY/716/2007	E10	6	160	96	96
B-Vic, B/BR/60/2008	E11	5	16	42	42

*GMT: geometric mean titer; Pre, pre-vaccination; Post, post-vaccination.

†The 2009–10 Northern Hemisphere egg-based trivalent influenza vaccine contains H1N1 A/Brisbane/59/2007-like (BR/59/2007), H3N2 A/Uruguay/716/2007-like (URY/716/2007), and B-Victoria lineage (B-Vic) B/Brisbane/60/2008 (B/BR/60/2008).

‡Percentage with post-vaccination HAI titer ≥40.

§Percentage with 4-fold increase of HAI titer when either a pre-vaccination HAI titer ≤1:10 and a post-vaccination HAI titer ≥1:40 or a pre-vaccination HAI titer >1:10 and a minimum 4-fold rise in post-vaccination HAI antibody titer.

Supplementary Table S4. Hemagglutination inhibition responses in healthy subjects administered 2010–11 Northern Hemisphere egg-based seasonal trivalent influenza vaccine against homologous vaccine viruses.

		Adults (n = 24), mean age 46.1 Yr (range, 18.4-64.0 Yr)			
		GMT*			
Homologous vaccine virus†	Passage	Pre	Post	% Seroprotection‡	% Seroconversion§
pH1N1, CA/07/2009	E6	5	69	67	67
H3N2, PE/16/2009	E5	5	18	29	29
B-Vic, B/BR/60/2008	E11	5	15	29	29

*GMT: geometric mean titer; Pre, pre-vaccination; Post, post-vaccination.

†The 2010–11 Northern Hemisphere egg-based trivalent influenza vaccine contains pandemic H1N1 (pH1N1) A/California/07/2009-like (CA/07/2009), H3N2 A/Perth/16/2009-like (PE/16/2009), and B-Victoria lineage (B-Vic) B/Brisbane/60/2008 (B/BR/60/2008).

‡Percentage with post-vaccination HAI titer ≥40.

§Percentage with 4-fold increase of HAI titer when either a pre-vaccination HAI titer ≤1:10 and a post-vaccination HAI titer ≥1:40 or a pre-vaccination HAI titer >1:10 and a minimum 4-fold rise in post-vaccination HAI antibody titer.

Supplementary Table S5. Hemagglutination inhibition responses of standard ferret post-infection antisera following intranasal infection of ferrets with egg-grown or cell-grown H3 viruses.*

Clade	Strain	Standard ferret post-infection antisera								
		Anti-URY/07e	Anti-URY/07c	Anti-VIC/11e	Anti-VIC/11c	Anti-TX/12e	Anti-TX/12c	Anti-NC/14e	Anti-NC/14c	Anti-PE/09e
1	URY/07e	1280	320	10	20	10	10	10	10	10
	PE/09e	10	20	160	640	160	320	40	40	640
3C.1	VIC/11e	20	40	1280	1280	1280	2560	320	160	640
	VIC/11c	40	80	80	160	160	320	80	40	160
	TX/12e	40	80	640	640	1280	1280	320	80	320
	TX/12c	20	40	40	40	160	160	40	20	80
3C.2	UT/13e	10	20	640	640	640	640	320	160	320
	UT/13c	40	80	40	40	160	160	80	40	160
	CRI/13e	80	80	80	640	320	320	160	160	40
	CRI/13c	40	40	40	80	160	160	80	40	80
3C.2a	MI/14c	5	80	80	320	160	160	160	40	40
3C.3a	SWZ/13e	10	10	80	80	80	320	320	160	20
	SWZ/13c	20	40	40	320	320	320	640	320	80
	PL/14e	10	10	40	640	80	640	1280	320	40
	NC/14e	10	10	160	160	160	1280	1280	160	40
	NC/14c	10	40	20	80	80	80	160	80	40

*Egg-grown H3N2 strains are A/Uruguay/716/2007 (NYMCX175C) (URY/07e), A/Perth/16/2009 (PE/09e), A/Victoria/361/2011 (VIC/11e), A/Texas/50/2012 (TX/12e), A/Costa Rica/4700/2013 (CRI/13e), A/Utah/07/2013 (UT/13e), A/Switzerland/9715293/2013 (SWZ/13e), A/Palau/6759/2014 (PL/14e), and A/North Carolina/13/2014 (NC/14e).
Cell-grown H3N2 viruses are A/Victoria/361/2011 (VIC/11c), A/Texas/50/2012 (TX/12c), A/Costa Rica/4700/2013 (CRI/13c), A/Utah/07/2013 (UT/13c), A/Switzerland/9715293/2013 (SWZ/13c), A/North Carolina/13/2014 (NC/14c), and A/Michigan/15/2014 (MI/14c).

Supplementary Table S6. Cross-reactive hemagglutination inhibition responses of post-vaccination adult sera* against recent influenza A (H3) isolates†.

Clade	Strain	Cross-reactive HAI Responses of Post-Vaccination Adult Sera (GMT)‡			
		2014–15 NH Egg-based Vaccine (n= 30)	2014–15 NH Cell-based Vaccine (n= 24)	2010–11 NH Egg-based Vaccine (n= 24)	2009–10 NH Egg-based Vaccine (n= 24)
1	URY/07e	20	29	19	160
	PE/09e	31	19	28	78
3C.1	VIC/11e	130	71	76	202
	VIC/11c	19	6	10	52
	TX/12e	76	34	42	65
	TX/12c	20	22	10	20
3C.2	UT/13e	80	41	39	57
	UT/13c	20	18	9	19
	CRI/13e	86	24	19	41
	CRI/13c	14	20	8	15
3C.2a	MI/14c	13	15	21	ND
3C.3a	SWZ/13e	34	18	24	60
	SWZ/13c	29	48	17	ND
	PL/14e	14	23	11	33
	NC/14e	22	15	16	27
	NC/14c	10	14	6	23

* Post-vaccination sera were collected from healthy adult subjects administered Northern Hemisphere (NH) cell-based vaccine for 2014–15 season, or NH egg-based vaccine for 2009–10, 2010–11 or 2014–15 season respectively.

† HAI: hemagglutination inhibition; GMT: geometric mean titer; ND: not determined due to limited volumes of human post-vaccination sera.

‡Egg-grown H3N2 strains are A/Uruguay/716/2007 (NYMCX175C) (URY/07e), A/Perth/16/2009 (PE/09e), A/Victoria/361/2011 (VIC/11e), A/Texas/50/2012 (TX/12e), A/Costa Rica/4700/2013 (CRI/13e), A/Utah/07/2013 (UT/13e), A/Switzerland/9715293/2013 (SWZ/13e), A/Palau/6759/2014 (PL/14e), and A/North Carolina/13/2014 (NC/14e).

Cell-grown H3N2 viruses are A/Victoria/361/2011 (VIC/11c), A/Texas/50/2012 (TX/12c), A/Costa Rica/4700/2013 (CRI/13c), A/Utah/07/2013 (UT/13c), A/Switzerland/9715293/2013 (SWZ/13c), A/North Carolina/13/2014 (NC/14c), and A/Michigan/15/2014 (MI/14c).

Supplementary Table S7. Amino acid differences in hemagglutinin antibody-binding sites of recent influenza A (H3N2) virus variants relative to homologous H3 vaccine viruses of the 2009–10, 2010–11, 2012–13, and 2014–15 Northern Hemisphere seasonal influenza vaccines*.

Virus†			Antibody-binding Site with HA1 Position (H3 Numbering) ‡																																		
			A					B										C								D							E				
Clade	Strain	Host	124	138	140	142	145	128	156	157	158	159	186	189	192	193	198	45	48	53	54	278	280	304	312	103	121	173	212	219	226	230	62	67	88	94	262
1	URY/07	egg	S	S	I	R	N	T	H	L	K	F	G	N	I	F	A	S	T	D	S	N	E	A	N	P	N	K	T	S	I	I	E	I	V	Y	S
	PE/09	egg	-	A	-	-	-	-	-	-	N	-	-	K	-	-	-	-	-	-	-	-	-	-	-	-	-	Q	-	-	-	-	K	-	-	-	-
3C.1	VIC/11	egg	-	A	-	-	-	-	Q	-	N	-	V	K	-	-	S	N	I	-	-	-	-	-	S	-	-	Q	A	Y	-	-	-	-	-	-	-
		cell	-	A	-	-	-	-	-	-	N	-	-	K	-	-	S	N	I	-	-	-	-	-	S	-	-	Q	A	-	-	-	-	-	-	-	-
	TX/12	egg	-	A	-	-	-	N	-	-	N	-	V	K	-	-	P	N	I	-	-	K	-	-	S	-	-	Q	A	F	-	-	-	-	-	-	-
		cell	-	A	-	-	-	N	-	-	N	-	-	K	-	-	P	N	I	-	-	K	-	-	S	-	-	Q	A	-	-	-	-	-	-	-	-
3C.2	CRI/13	egg	-	A	-	-	S	-	-	-	N	-	-	K	-	-	S	N	I	-	-	K	-	-	S	-	-	Q	A	-	-	-	-	-	-	H	-
		cell	-	A	-	-	S	-	-	-	N	-	-	K	-	-	S	N	I	-	-	K	-	-	S	-	-	Q	A	-	-	-	-	-	-	H	-
	UT/13	egg	-	A	-	G	S	A	-	S	N	-	V	K	-	-	S	N	I	-	-	K	-	-	S	-	-	Q	A	Y	-	-	-	-	-	-	-
		cell	-	A	-	G	S	A	-	S	N	-	-	K	-	-	S	N	I	-	-	K	-	-	S	-	-	Q	A	-	-	-	-	-	-	-	-
3C.2a	MI/14	cell	-	-	-	-	S	-	-	-	N	D	-	K	-	-	S	N	I	-	-	K	-	-	S	-	-	Q	A	-	-	-	-	-	-	-	-
3C.3a	SWI/13	egg	-	-	R	G	S	A	-	-	N	D	V	K	-	-	S	N	I	-	-	K	-	-	S	-	-	Q	A	X	-	-	-	-	-	-	-
		cell	-	-	-	G	S	A	-	-	N	D	-	K	-	-	S	N	I	-	-	K	-	-	S	-	-	Q	A	-	-	-	-	-	-	-	-
	PL/14	egg	-	-	-	G	S	A	-	-	N	G	-	K	-	-	S	N	I	-	-	K	-	-	S	-	-	Q	A	-	-	-	-	-	-	-	-
	NC/14	egg	-	-	K	G	S	A	-	-	N	D	V	K	-	-	S	N	I	-	-	K	-	-	S	-	-	Q	A	-	-	-	-	-	-	-	-
		cell	-	A	-	G	S	A	-	-	N	D	-	K	-	-	S	N	I	-	-	K	-	-	S	-	-	Q	A	-	-	-	-	-	-	-	-

*HA1: hemagglutinin 1.

†H3N2 strains are A/Uruguay/716/2007 (NYMCX175C) (URY/07), A/Perth/16/2009 (PE/09), A/Victoria/361/2011 (VIC/11), A/Texas/50/2012 (TX/12), A/Costa Rica/4700/2013 (CRI/13), A/Utah/07/2013 (UT/13), A/Switzerland/9715293/2013 (SWZ/13), A/Palau/6759/2014 (PL/14), A/North Carolina/13/2014 (NC/14), and A/Michigan/15/2014 (MI/14).

‡– indicates no change.

Supplementary Figure S1. Cross-reactive hemagglutination inhibition (HAI) responses of 2009–10, 2010–11, and 2014–15 Northern Hemisphere (NH) seasonal influenza vaccines against recent influenza A (H3) viruses. Sera were collected from healthy adults immunized with 2009–10, 2010–11, and 2014–15 NH egg-based or cell-based vaccine. Post-vaccination (post-vac) HAI titers against the H3 vaccine prototype virus and representative strains of recent H3N2 isolates were determined by using 1% guinea pig erythrocytes. There were 24 post-vac sera each from adults vaccinated with 2009–10 and 2010–11 NH egg-based vaccines and 2014–15 NH cell-based vaccine. There were also 30 post-vac sera from adults vaccinated with 2014–15 NH egg-based vaccine. The H3 strains in the testing panel included egg-grown A/Uruguay/716/2007 (NYMCX175C) (URY/07e), A/Perth/16/2009 (PE/09e), A/Victoria/361/2011 (VIC/11e), A/Costa Rica/4700/2013 (CRI/13e), A/Utah/07/2013 (UT/13e), and cell-grown A/Victoria/361/2011 (VIC/11c), A/Costa Rica/4700/2013 (CRI/13c), and A/Utah/07/2013 (UT/13c). The proportions of subjects with post-vac HAI titer of ≥40, ≥80 and ≥160 were plotted. [$]Indicates the H3 prototype virus of 2009–10 egg-based vaccine. [&]Indicates the H3 prototype virus of 2010–11 egg-based vaccine. ND: not determined due to limited volumes of sera. Dotted horizontal line indicates 50% achievement.

Supplementary Fig. S1

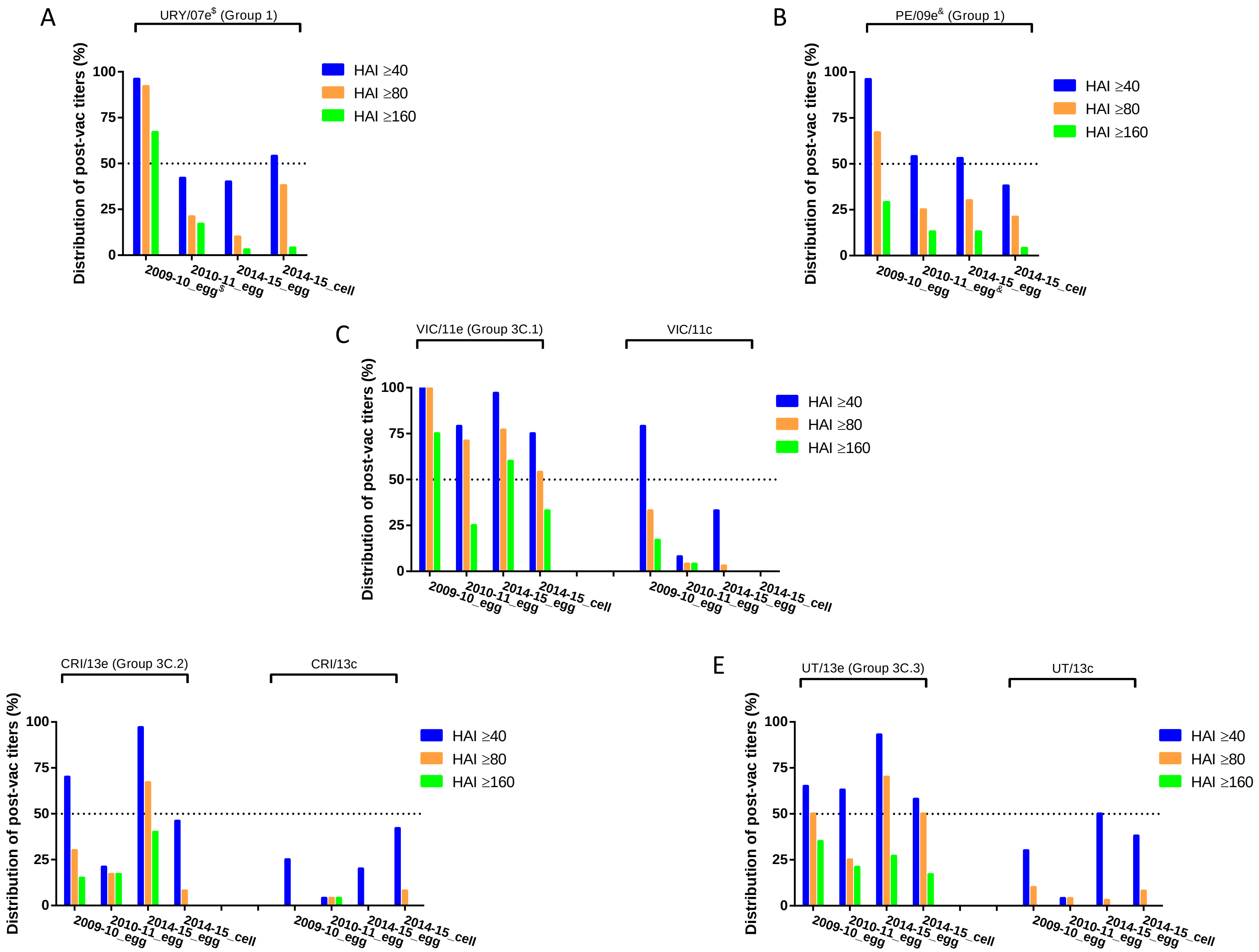

Supplement: Supplementary Information [file srep15279-s1.pdf]
